# Enzymatic production of *trans*‐4‐hydroxy‐l‐proline by proline 4‐hydroxylase

**DOI:** 10.1111/1751-7915.13616

**Published:** 2020-07-03

**Authors:** Xiulai Chen, Juyang Yi, Jia Liu, Qiuling Luo, Liming Liu

**Affiliations:** ^1^ State Key Laboratory of Food Science and Technology Jiangnan University Wuxi 214122 China; ^2^ Key Laboratory of Industrial Biotechnology Ministry of Education Jiangnan University Wuxi 214122 China; ^3^ International Joint Laboratory on Food Safety Jiangnan University Wuxi 214122 China; ^4^ Shaoxing Baiyin Biotechnology Co. Ltd Shaoxing 312000 China

## Abstract

*Trans*‐4‐hydroxy‐l‐proline (Hyp) is a useful chiral building block for production of many nutritional supplements and pharmaceuticals. However, it is still challenging for industrial production of Hyp due to heavy environmental pollution and low production efficiency. To establish a green and efficient process for Hyp production, the proline 4‐hydroxylase (*Ds*P4H) from *Dactylosporangium* sp. RH1 was overexpressed and functionally characterized in *Escherichia coli* BL21(DE3). The recombinant *Ds*P4H with l‐proline as a substrate exhibited *K*_m_, *k*_cat_ and *k*_cat_/*K*_m_ values up to 0.80 mM, 0.52 s^−1^ and 0.65 s^−1^·mM^−1^ respectively. Furthermore, *Ds*P4H showed the highest activity at 35°C and pH 6.5 towards l‐proline. The highest enzyme activity of 175.6 U mg^−1^ was achieved by optimizing culture parameters. Under the optimal transformation conditions in a 5‐l fermenter, Hyp titre, conversion rate and productivity were up to 99.9 g l^−1^, 99.9% and 2.77 g l^−1^ h^−1^ respectively. This strategy described here provides an efficient method for production of Hyp and thus has a great potential in industrial application.

## Graphical abstract

*Trans*‐4‐hydroxy‐l‐proline (Hyp) is a useful chiral building block for production of many nutritional supplements and pharmaceuticals. Here, the proline 4‐hydroxylase (DsP4H) from *Dactylosporangium* sp. RH1 was overexpressed and functionally characterised in *Escherichia coli* BL21(DE3) to improve enzymatic production of Hyp. Under the optimal transformation conditions in a 5‐l fermenter, Hyp titer, conversion rate, and productivity were up to 99.9 g l^−1^, 99.9%, and 2.77 g l^−1^ h^−1^, respectively.

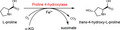

## Introduction

*Trans*‐4‐hydroxy‐l‐proline (Hyp), one of the hydroxyproline isomers, is a useful chiral building block, which can be used as nutritional supplements in food industry and as intermediate in pharmaceutical industry (Bach and Takagi, 2013; Houwaart *et al*., 2014). Currently, there are three approaches for Hyp production: chemical synthesis (Zhao *et al*., 2017), microbial fermentation (Zhang *et al*., 2018) and enzymatic transformation (Shibasaki *et al*., 2000b). Although chemical synthesis has been used to produce Hyp on a large scale, it has many serious obstacles such as low recovery rate and heavy environmental pollution (Liu *et al*., 2019). Although microbial fermentation has made great progress, its production efficiency is low (Zhang *et al*., 2018). Given these shortcomings, considerable interest has been shown in enzymatic transformation for producing Hyp, which is regarded as a promising method due to its high catalytic efficiency and environmental compatibility (Shibasaki *et al*., 2000b; Zhao *et al*., 2017).

Proline 4‐hydroxylase (P4H) has great potential to be used for the production of Hyp. P4H was initially discovered for hydroxylation of l‐proline to Hyp in *Streptomyces griseoviridus* (Onishi *et al*., 1984). When P4H from *Dactylosporangium* sp. RH1 (*Ds*P4H) was expressed in *Escherichia coli* BL21(DE3) and *Corynebacterium glutamicum*, the recombinant *E. coli* and *C. glutamicum* strains could produce Hyp in the presence of l‐proline and α‐ketoglutarate (α‐KG) (Yi *et al*., 2014). To improve production efficiency of Hyp, P4Hs from various microorganisms were expressed in different *E. coli* strains. Hyp titre (15.72 g l^−1^) was improved by expressing α‐KG‐dependent dioxygenase from *Kutzneria albida* (*Ka*PH1) in *E. coli* BL21(DE3) (Jing *et al*., 2019). When P4H from *uncultured bacterium* esnapd13 (*Ub*P4H) was engineered to simultaneously improve its activity and thermostability by loop grafting and site‐directed mutagenesis, the best mutant *Ub*P4H‐Da‐E112P in *E. coli* MG1655Δ*putA* was able to produce 12.9 g l^−1^ Hyp (Liu *et al*., 2019). Similarly, *Ds*P4H was introduced into *E. coli* W1485Δ*putA*, and the final concentration of Hyp was up to 41 g l^−1^ with its yield 87% (Shibasaki *et al*., 2000b). Further, Hyp production (45.83 g l^−1^) was largely enhanced by expressing P4H from *Alteromonas mediterranea* (*Al*P4H) and a γ‐glutamyl kinase (proB) mutation in *E. coli* MG1655Δ*putA* (Falcioni *et al*., 2015). The above research results have indicated that Hyp production can be successfully improved by enzymatic transformation. However, Hyp could not be synthesized efficiently by converting l‐proline under one‐step catalysis of P4H. Hence, it is still challenging for developing Hyp‐producing strains with excellent catalytic performance.

**Fig. 1:**
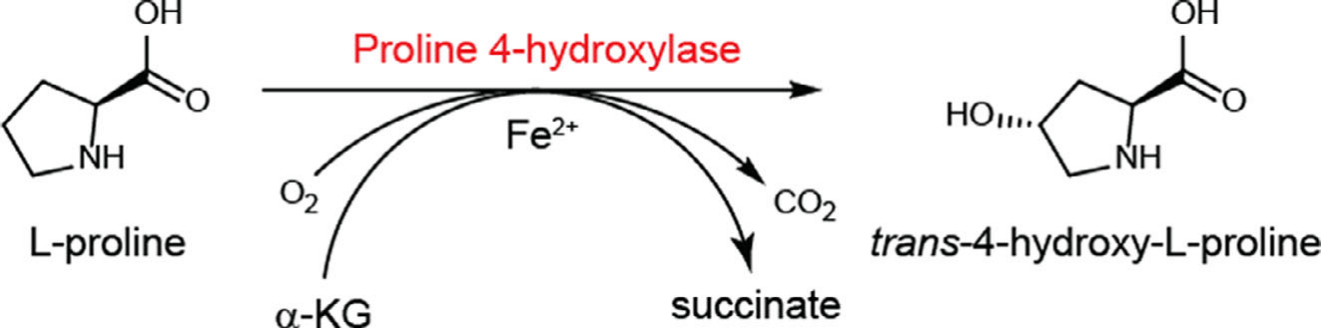
Enzymatic production of *trans*‐4‐hydroxy‐l‐proline by proline 4‐hydroxylase in *E. coli*. α‐KG: α‐ketoglutarate.

In this study, we expressed and characterized the proline 4‐hydroxylase from *Dactylosporangium* sp. RH1 and then constructed a whole‐cell biocatalyst by optimizing culture parameters and conversion conditions for the biosynthesis of Hyp from l‐proline (Fig. 1). The final concentration of Hyp reached 99.9 g l^−1^ from 100 g l^−1^ l‐proline in 36 h by one‐step process, which substantially enhanced the efficiency of Hyp production.

## Results

### Screening and expression of P4H in *E. coli*

**Fig. 2:**
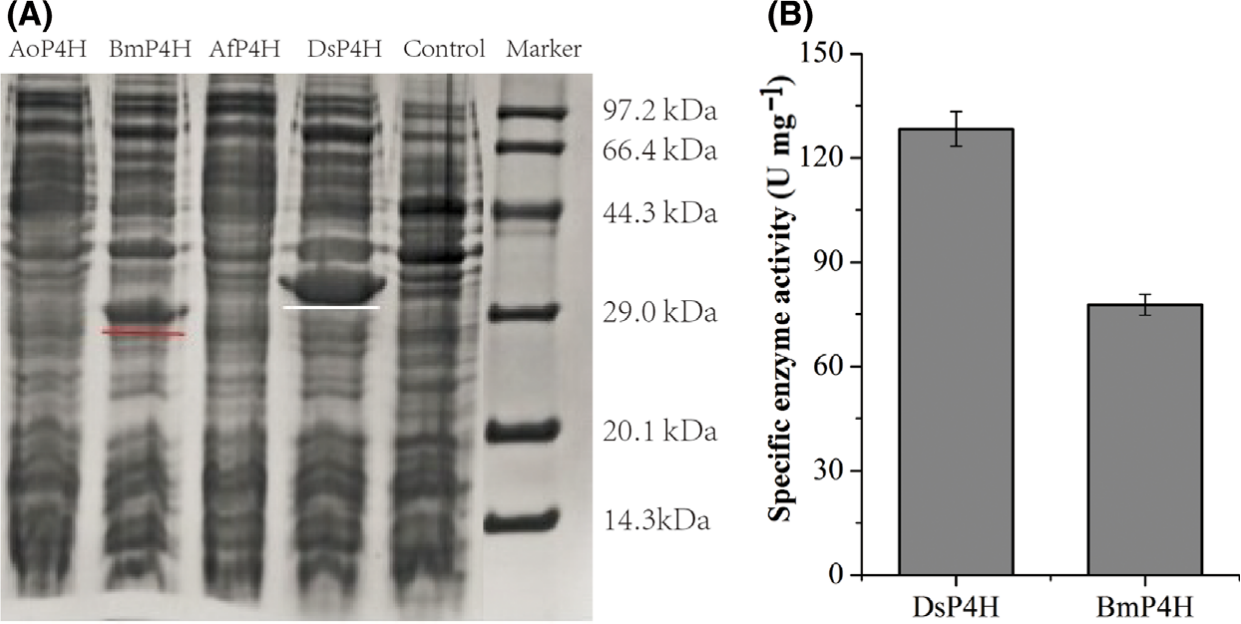
Expression of P4Hs and their catalytic activities on l‐proline. A. SDS‐PAGE analysis of the recombinant P4H proteins. B. The specific enzyme activity of *Ds*P4H and *Bm*P4H.

Proline 4‐hydroxylase (P4H) from *Dactylosporangium* sp. RH1 (*Ds*P4H) has been used for enzymatic production of *trans*‐4‐hydroxy‐l‐proline (Hyp) with l‐proline as substrates (Shibasaki *et al*., 2000b). Thus, *Ds*P4H was chosen as a probe sequence to screen potential enzymes in UniProt database for converting l‐proline to Hyp, and three P4Hs from *Bacillus megaterium* (*Bm*P4H), *Aspergillus oryzae* (*Ao*P4H) and *Aspergillus flavus* (*Af*P4H) were selected. These genes were inserted into the expression plasmid pET28a for its overexpression in *E. coli* BL21(DE3), respectively, and the activities of the selected enzymes were assayed. We observed that only *Ds*P4H and *Bm*P4H were expressed in soluble protein (Fig. 2A), and *Ds*P4H exhibited higher enzyme activity (128.3 U mg^−1^), which was 64.8% higher than that of *Bm*P4H (77.8 U mg^−1^) under the same conditions (Fig. 2B). Therefore, *Ds*P4H was selected for further research.

### Characterization of recombinant *Ds*P4H

**Fig. 3:**
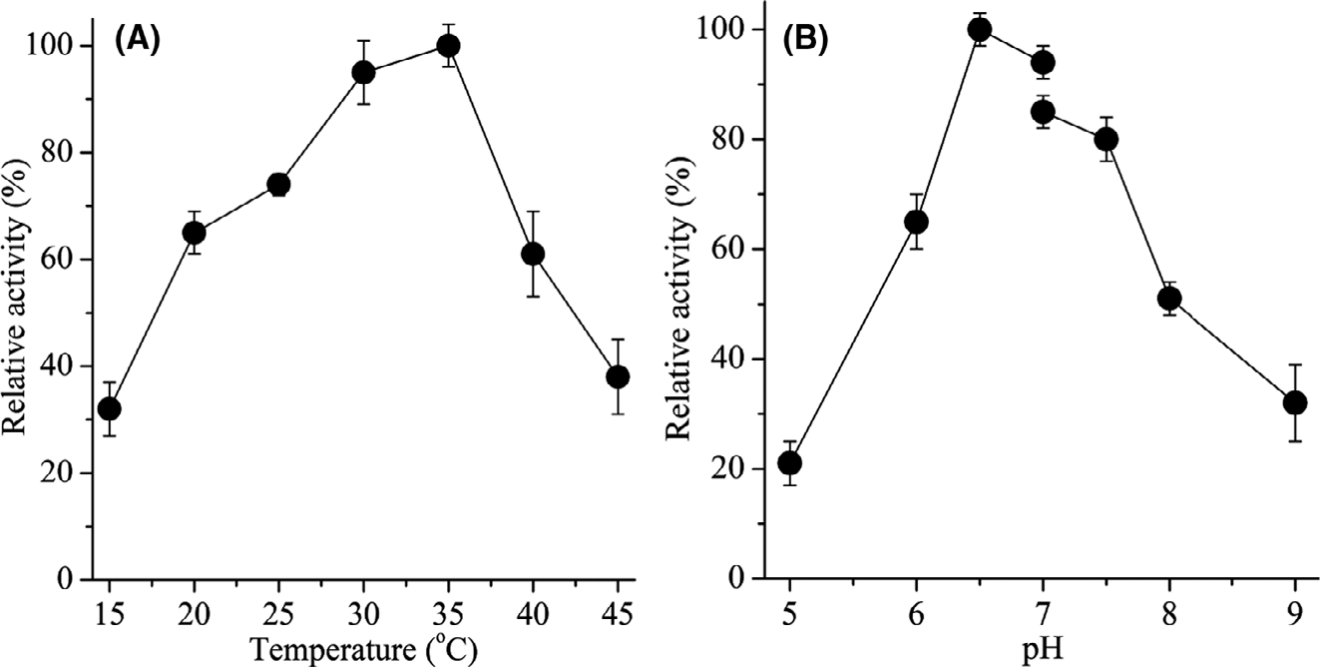
Effect of temperature and pH on the catalytic activity of recombinant *Ds*P4H. A. The optimal temperature of l‐proline hydroxylation by *Ds*P4H. B. The optimal pH of l‐proline hydroxylation by *Ds*P4H. The following buffers were used: MES‐Tris buffer (0.05 M, pH 5.0–7.0), Tris‐HCl buffer (0.05 M, pH 7.0–9.0). The maximal enzyme activity of *Ds*P4H was set to 100%.

The recombinant *Ds*P4H was purified by HisTrap™ HP affinity column, and the effects of temperature (15–45°C) and pH (4.0–9.0) on its catalytic activity were investigated respectively. The relative activity of recombinant *Ds*P4H increased with increasing temperature from 15 to 35°C and decreased from 35 to 45°C (Fig. 3A). The maximum activity of the recombinant *Ds*P4H was observed at 35°C (Fig. 3B). The relative activity of the recombinant *Ds*P4H was maintained above 60% at a pH range of 6.0–8.0 and reached the maximum activity at pH 6.5 (Fig. 3B). These results indicated that the optimal temperature and pH for the catalytic activity of recombinant *Ds*P4H were 35°C and pH 6.5 respectively.

Based on this, the kinetic parameters of *Ds*P4H were measured by varying l‐proline and α‐ketoglutarate (α‐KG) concentrations. For *Ds*P4H with l‐proline as a substrate, the *K*_m_, *k*_cat_ and *k*_cat_/*K*_m_ values were 0.80 mM, 0.52 s^−1^ and 0.65 s^−1^ mM^−1^ respectively (Table 1). The catalytic efficiency of *Ds*P4H (*k*_cat_/*K*_m_) was 27.5% and 17.4‐fold higher than that of *Ka*PH1 and *Ub*P4H respectively (Table 1). For *Ds*P4H with α‐KG as a substrate, the *K*_m_, *k*_cat_ and *k*_cat_/*K*_m_ values were 1.08 mM, 0.62 s^−1^ and 0.58 s^−1^ mM^−1^ respectively (Table 1). The catalytic efficiency of *Ds*P4H (*k*_cat_/*K*_m_) was similar to that of *Ka*PH1 (Table 1). These results showed that *Ds*P4H would be favourable for l‐proline hydroxylation that involves α‐KG decarboxylation.

**Table 1:** The kinetic parameters of recombinant *Ds*P4H with l‐proline and α‐KG as substrates.

Enzyme	Substrate	*K*_m_ (mM)	*k*_cat_ (s^−1^)	*k*_cat_/*K*_m_ (s^−1^ mM^−1^)	Reference
*Ds*P4H	l‐proline	0.80 ± 0.03	0.52 ± 0.01	0.65	This study
	α‐KG	1.08 ± 0.05	0.62 ± 0.02	0.58	
*Ka*PH1	l‐proline	1.07 ± 0.11	0.54 ± 0.01	0.51 ± 0.05	Jing *et al*. (2019)
	α‐KG	0.84 ± 0.10	0.50 ± 0.02	0.59 ± 0.05	
*Ub*P4H	l‐proline	0.68 ± 0.07	0.0238 ± 0.0007	0.0353	Liu *et al*. (2019)
	α‐KG	–	–	–	

### Improving expression of recombinant *Ds*P4H

**Fig. 4:**
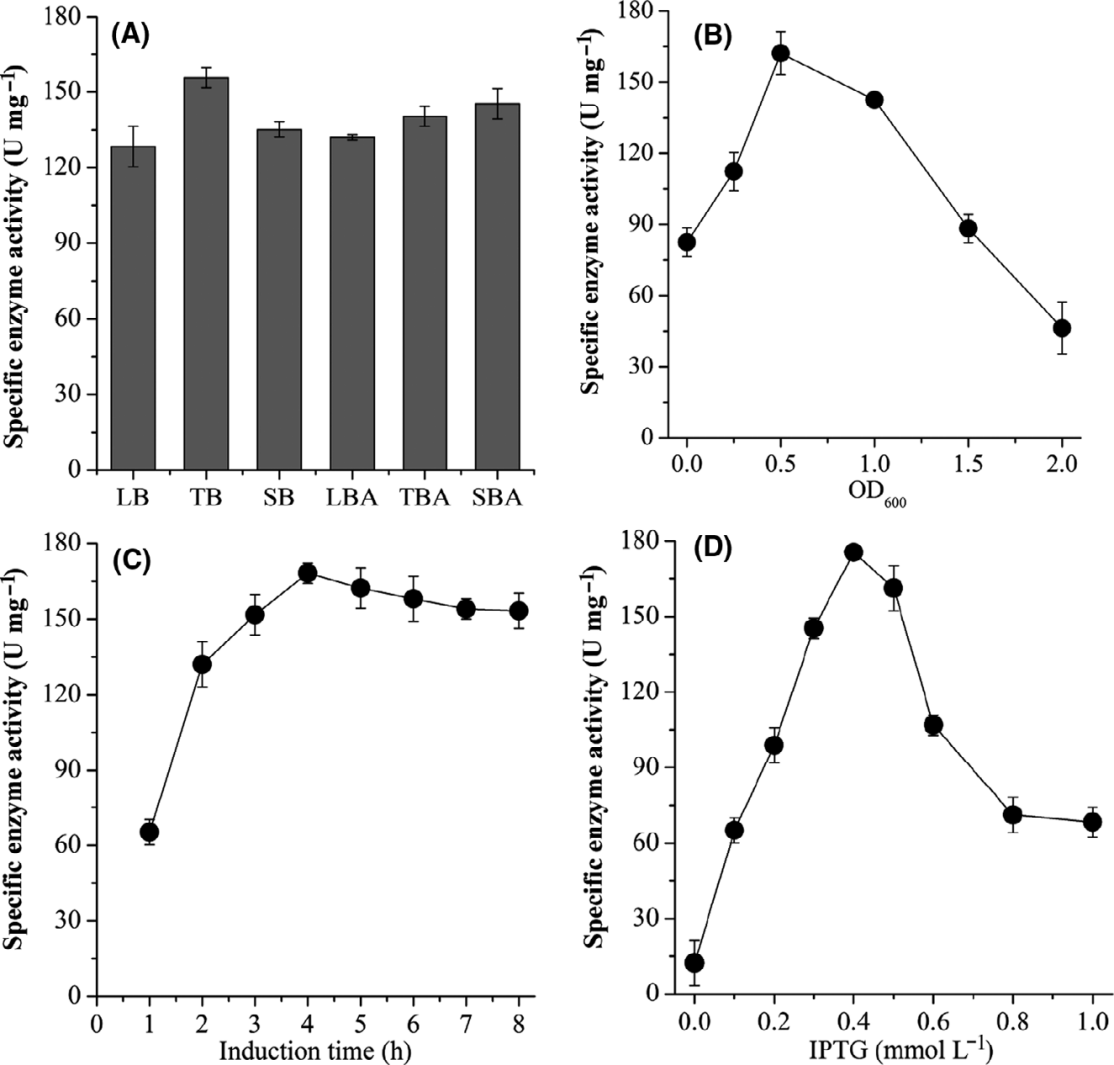
Effect of culture parameters on the production of recombinant *Ds*P4H. A. Medium. B. Induction phase. C. Induction time. D. IPTG concentration.

To maximize overexpression of recombinant *Ds*P4H, many culture parameters were investigated. *E. coli* BL21‐*Ds*P4H was cultured in different medium such as LB, TB, SB, LBA, TBA and SBA. *Ds*P4H activity reached its maximum value up to 155.6 U mg^−1^, when TB was used as culture medium (Fig. 4A). *Ds*P4H activity was increased by 21.3% compared with that of LB medium. Then, induction phase was tested, and the highest *Ds*P4H activity (162.2 U mg^−1^) was achieved with an induction during the exponential growth phase (OD_600_ = 0.5) (Fig. 4B). After this stage, induction did not lead to any significant increase in *Ds*P4H activity (Fig. 4B). Next, induction time was investigated, and at least 4 h induction was required to obtain a maximum *Ds*P4H activity (168.2 U mg^−1^) (Fig. 4C). Finally, the concentration of inducer was optimized, and the maximum recombinant *Ds*P4H activity (175.6 U mg^−1^) was observed with 0.4 mmol l^−1^ IPTG (Fig. 4D). Thus, the optimal parameters for recombinant *Ds*P4H production were TB medium, exponential phase (OD_600_ = 0.5), induction time at least 4 h, and 0.4 mmol l^−1^ IPTG.

### Optimizing whole‐cell biotransformation from l‐proline to Hyp

**Fig. 5:**
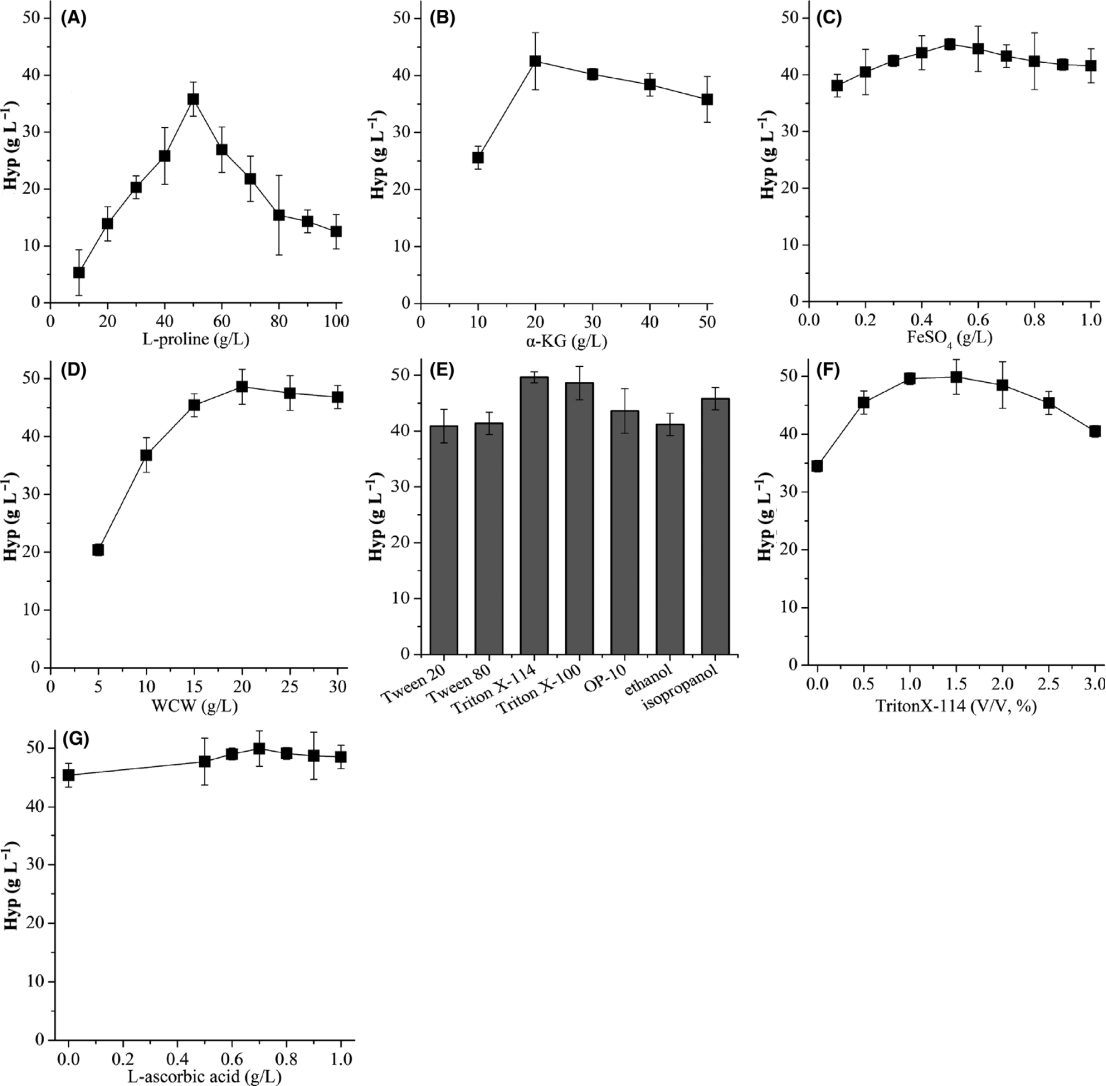
Effect of conversion conditions on Hyp production. A. l‐proline concentration. B. α‐KG concentration. C. FeSO_4_ concentration. D. Wet cell weight of intact cells. E. Permeability reagents. F. Triton X‐114 concentration. G. l‐ascorbic acid concentration.

Since recombinant *Ds*P4H is an intracellular protein, *E. coli* BL21‐*Ds*P4H cells need to be harvested by centrifugation before it is used for biotransformations. In order to convert l‐proline to Hyp efficiently, the optimal conversion conditions were determined. We firstly analysed the effect of different concentrations (10–100 g l^−1^) of l‐proline on Hyp production. The highest Hyp concentration was achieved up to 35.8 g l^−1^ from 50 g l^−1^ l‐proline (Fig. 5A). The Hyp conversion rate was increased with the increase of l‐proline concentration from 10 to 50 g l^−1^, but no further increase was observed with > 50 g l^−1^ l‐proline (Fig. 5A). When α‐KG concentration was varied from 10 to 50 g l^−1^, Hyp production peaked at 42.5 g l^−1^ from 50 g l^−1^ l‐proline with 20 g l^−1^ α‐KG, and Hyp conversion rate reached 85.0% (Fig. 5B). Since exogenous Fe^2+^ ions were required for *Ds*P4H activity, 0.1–1.0 g l^−1^ FeSO_4_ was tested, and 0.5 g l^−1^ FeSO_4_ was found to be optimal for Hyp production (45.4 g l^−1^) (Fig. 5C). The above results indicated that the optimal concentration of substrates for Hyp production was as follows: 50 g l^−1^ l‐proline, 20 g l^−1^ α‐KG and 0.5 g l^−1^ FeSO_4_.

To further improve Hyp production, the effects of wet cell weight (WCW), permeability reagents and l‐ascorbic acid were investigated respectively. When WCW was changed from 5 to 30 g l^−1^, the maximum production of Hyp reached 48.6 g l^−1^ from 50 g l^−1^ l‐proline with 20 g l^−1^ WCW, and Hyp conversion rate was increased to 97.2% (Fig. 5D). Then, several permeability reagents (1 (v/v) % Triton X‐114, Triton X‐100, OP‐10, ethanol, isopropanol, Tween 20 and Tween 80) were used to treat 20 g l^−1^ WCW for 30 min respectively. Triton X‐114, Triton X‐100 and Tween 80 showed positive effect on Hyp production, but OP‐10, ethanol, isopropanol and Tween 20 displayed negative effect (Fig. 5E). The highest production of Hyp (49.6 g l^−1^) was obtained with Triton X‐114 addition (Fig. 5E). Based on this, we checked the effect of Triton X‐114 addition on Hyp production. As shown in Figure 5F, the optimal concentration of Triton X‐114 was 1.5 (v/v) %. Finally, we tested the effect of different concentrations (0–1.0 g l^−1^) of l‐ascorbic acid on Hyp production and found that 0.7 g l^−1^ l‐ascorbic acid was optimal for Hyp production (Fig. 5G). Under the optimal conditions, the highest titre of Hyp reached 49.9 g l^−1^ from 50 g l^−1^ l‐proline with Hyp conversion rate 99.8% (Fig. 5G). These results showed that the optimal WCW, permeability reagents and l‐ascorbic acid were 20 g l^−1^ WCW, 1.5 (v/v) % Triton X‐114 and 0.7 g l^−1^ l‐ascorbic acid respectively.

### Producing Hyp with *E. coli* BL21‐*Ds*P4H in a 5‐l fermentor

**Fig. 6:**
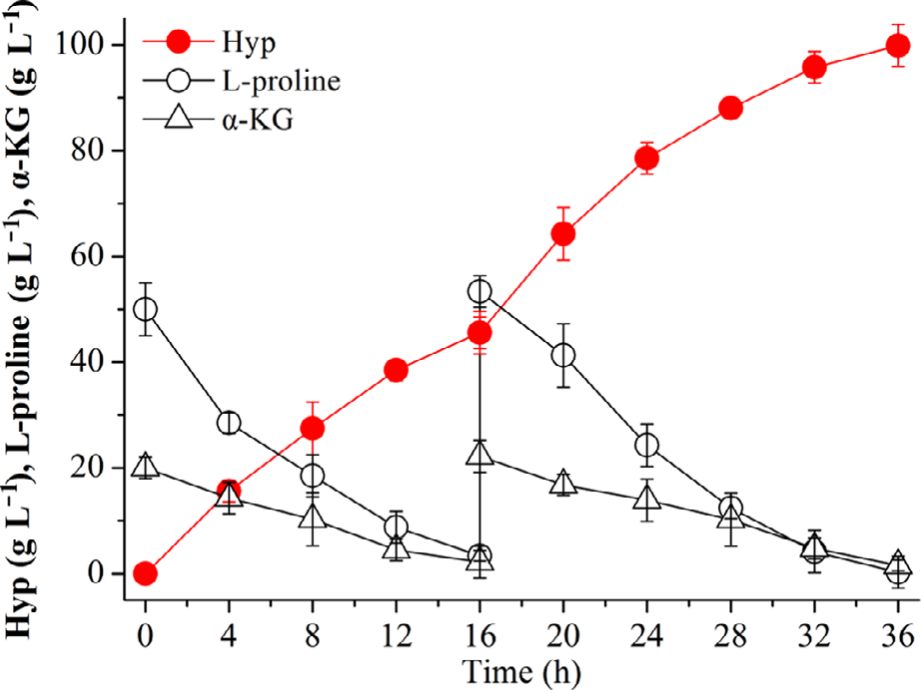
Hyp production with *E. coli* BL21‐*Ds*P4H under the optimal conversion conditions in a 5‐l bioreactor.

Based on the above experiments, we further explored the potential of whole‐cell biocatalyst of the recombinant strain *E. coli* BL21‐*Ds*P4H for the transformation of l‐proline to Hyp in a 5‐l bioreactor. In this transformation process, l‐proline and α‐KG were rapidly consumed during Hyp synthesis (Fig. 6). Hyp accumulated gradually in the broth from 0 to 36 h, and the final Hyp titre, conversion rate and productivity were up to 99.9 g l^−1^, 99.9% and 2.77 g l^−1^ h^−1^ respectively (Fig. 6). However, the productivity of Hyp in 0–16 h was higher than that in 16–36 h (Fig. 6), possibly due to the decreased enzyme efficiency. These results indicated that *E. coli* BL21‐*Ds*P4H was useful for scale‐up culture, suggesting that it has great potential for industrial production of Hyp in the future.

## Discussion

In this study, to establish a green and efficient process for Hyp production, the *Ds*P4H from *Dactylosporangium* sp. RH1 was overexpressed in *E. coli* BL21 (DE3), and 175.6 U mg^−1^ recombinant *Ds*P4H activity was achieved in TB medium under the optimal culture parameters. To convert l‐proline to Hyp efficiently, conversion conditions were optimized, and the highest titre of Hyp reached 49.9 g l^−1^ with conversion rate 99.8%. Under these optimal transformation conditions, the final Hyp titre, conversion rate and productivity in a 5‐l fermenter were up to 99.9 g l^−1^, 99.9% and 2.77 g l^−1^ h^−1^ respectively. This is an efficient bioprocess for Hyp production, which has a great potential in industrial application.

Enzymatic transformation for Hyp production has many advantages over chemical synthesis and microbial fermentation. In this study, Hyp biosynthesis from L‐proline can be catalysed by P4H with α‐KG and oxygen as co‐substrates to generate succinate and CO_2_ in the presence of ferrous ion (Lawrence *et al*., 1996). In this process of whole‐cell transformation, the intracellular enzymes are often more stable in a protected environment, and thus, loss of enzyme activity is kept to a minimum (Song *et al*., 2015). These characteristics indicate that the enzymatic production of Hyp described in this study is more simple and environment‐friendly than that of chemical synthesis (Zhao *et al*., 2017). However, there are still many problems to be resolved before industrial production of Hyp, such as production costs. In this study, l‐proline, α‐KG, FeSO_4_, IPTG, LB and TB medium are used for enzymatic production of Hyp, which can increase the potential costs of Hyp production. To lower its costs, further stepwise improvement may mainly centre on five strategies. (i) Constructing l‐proline‐producing *E. coli* for Hyp production (Shibasaki *et al*., 2000a,b). Based on this, Hyp production can be achieved by two steps, that is, the first step is used for l‐proline production by microbial fermentation, and the second step is used for Hyp production by enzymatic transformation. (ii) Engineering α‐KG‐producing *E. coli* for enzymatic production of Hyp. Currently, the engineered strain *E. coli* 0901 has been applied for converting l‐proline to Hyp without α‐KG addition, but Hyp production was only up to 49.8 g l^−1^ (Chen *et al*., 2020). (iii) Replacing IPTG with lactose as induction agents. Lactose has been used for enzymatic production of α‐KG in our previous study (Fan *et al*., 2016). (iv) Using a cheaper media or simple salt media. For example, yeast extract and peptone in LB and TB medium can be replaced with corn steep liquor powder. (v) Reducing the formation of by‐products. In this study, *E. coli* BL21‐*Ds*P4H could efficiently convert 100 g l^−1^ l‐proline to 99.9 g l^−1^ Hyp, but at the same time 8.8 g l^−1^ succinate was formed. This accumulation of succinate is not beneficial to the subsequent product separation, extraction and purification (Hausinger, 2004).

Enzymatic transformation could enhance the ability effectively to convert l‐proline to Hyp. This is efficient for improving Hyp productivity. For one thing, when *E. coli* SEcH(pTc‐B74A‐alp4h) was used for Hyp fermentation with glucose as substrates, Hyp titre was up to 45.38 g l^−1^ with its productivity 1.27 g l^−1^ h^−1^ (Wang *et al*., 2018). In this study, Hyp production reached 99.9 g l^−1^ with its productivity 2.77 g l^−1^ h^−1^ by enzymatic transformation. This study showed a 120.1% increase in Hyp titre and productivity compared with that of previous study. For another thing, when *E. coli* 3ΔW3110/pTrc99a‐*p4hy*‐*proba* was applied for fermentative production of Hyp with glucose as substrates, Hyp titre was increased to 30.0 g l^−1^ in 52 h (Zhang *et al*., 2018). In this study, Hyp production was up to 99.9 g l^−1^ in 36 h by whole‐cell transformation. This result indicated that Hyp production cycle time in whole‐cell transformation is shortened by 44.4% compared with that of microbial fermentation. Thus, this study described here lays a good foundation for industrial production of Hyp in the future. Further, Hyp biosynthesis from l‐proline by enzymatic transformation not only solves overcapacity of l‐proline, but also provides an efficient approach to produce Hyp.

## Experimental procedures

### Strains and plasmids

The host strain *E. coli* BL21(DE3) and expression vector pET28a were purchased from Novegen. All strains and plasmids used in this study were listed in Table 2.

**Table 2:** Strains and plasmids used in this study.

Strains and plasmids	Relevant characteristics	References
Strains		
*E. coli* BL21(DE3)	F^−^ *ompT hsdS_B_* (*r_B_* ^−^ *m_B_* ^−^) *gal dcm* (DE3)	Novagen
*E. coli* BL21‐*Ds*P4H	*E. coli* BL21(DE3) (pET28a‐*Ds*P4H)	This study
*E. coli* BL21‐*Bm*P4H	*E. coli* BL21(DE3) (pET28a‐*Bm*P4H)	This study
*E. coli* BL21‐*Ao*P4H	*E. coli* BL21(DE3) (pET28a‐*Ao*P4H)	This study
*E. coli* BL21‐*Af*P4H	*E. coli* BL21(DE3) (pET28a‐*Af*P4H)	This study
Plasmids		
pET28a	ColE1, *Kan*, P_T7_	Novagen
pET28a‐*Ds*P4H	ColE1, *Kan*, P_T7_‐*Ds*P4H	This study
pET28a‐*Bm*P4H	ColE1, *Kan*, P_T7_‐*Bm*P4H	This study
pET28a‐*Ao*P4H	ColE1, *Kan*, P_T7_‐*Ao*P4H	This study
pET28a‐*Af*P4H	ColE1, *Kan*, P_T7_‐*Af*P4H	This study

### DNA manipulation

Proline 4‐hydroxylase (P4H) gene from *Dactylosporangium* sp. RH1 (*Ds*P4H, Gene ID: D78338.1) was artificially synthesized with codon optimization by Shanghai Sunny Biotechnology. *Bm*P4H gene (Gene ID: BMWSH_2348) was amplified from the chromosomal DNA of *Bacillus megaterium* WSH‐002. P4H genes from *Aspergillus oryzae* RIB40 (*Ao*P4H, Gene ID: AOR_1_1350154) and *Aspergillus flavus* NRRL3357 (*Af*P4H, Gene ID: AFLA_030540) were amplified with the corresponding *c*DNA as a template respectively. Then, the purified DNA fragments were digested with restriction enzymes and ligated into expression vector pET28a. Next, the verified recombinant plasmids were transformed into *E. coli* BL21(DE3) competent cells.

### Medium

Lysogeny broth (LB) medium used for seed cultures: 5 g l^−1^ yeast extract, 10 g l^−1^ peptone, 5 g l^−1^ NaCl. Kanamycin (100 mg ml^−1^) was added appropriately when needed.

Terrific broth (TB) medium used for fermentation in shake flasks: glycerol 5 g l^−1^, yeast extract 24 g l^−1^, tryptone 12 g l^−1^, KH_2_PO_4_ 2.31 g l^−1^, K_2_HPO_4_ 12.54 g l^−1^. Kanamycin (100 mg ml^−1^) and IPTG (0.4 mmol l^−1^) were added appropriately when needed.

LB auto‐induction medium (LBA), TB auto‐induction medium (TBA), Super broth (SB) and Super broth auto‐induction medium (SBA) were used as previously reported by Song *et al*. (2015). Kanamycin (100 mg ml^−1^) and IPTG (0.4 mmol l^−1^) were added appropriately when needed.

### Culture conditions

The seed cultures inoculated from a slant were cultivated on a reciprocal shaker (200 r.p.m.) at 37°C in a 250 ml flask containing 25 ml LB medium for 12 h. The seed cultures were then inoculated in 500 ml flasks containing 50 ml TB medium for shake flask fermentation at 37°C with rotation at 200 r.p.m. Cultures were induced when the optical density at 600 nm (OD_600_) was up to 0.5. This induction was continued for 4 h. After this, cell cultures were centrifuged and then washed by MES buffer (80 mM, pH 6.5). The obtained cells were used for the following biotransformation.

### Analytical methods

The optical density at 600 nm was measured using a spectrophotometer. The concentration of α‐KG was determined by high‐performance liquid chromatography (HPLC) (Zhang *et al*., 2009). l‐proline and Hyp were assayed by HPLC with a Zorbax Eclipse XDB‐C_18_ column (Agilent) at 40°C after derivatization with 2,4‐dinitrofluorobenzene (Zhang *et al*., 2019).

Conversion rate was determined using the following equation:

$$\text{Conversion}\;\text{rate}\;\left(\%\right)=\frac{M3}{M1-M2}\times 100$$

where *M*1 is the concentration of L‐proline before conversion, *M*2 is the remaining concentration of l‐proline after conversion, and *M*3 is the concentration of Hyp.

### Purification of P4H

The culture broth was centrifuged at 8000 × *g* for 5 min. The intact cells were suspended in 0.1 M phosphate buffer (pH 7.0) and sonicated by Ultrasonic Cell Disruptor. *Ds*P4H purification was performed as previously described by (Jing *et al*., 2019).

### Determination of kinetic parameters

The kinetic parameters (*V*_max_, *K*_m_, *k*_cat_ and *k*_cat_ */K*_m_) of *Ds*P4H were measured in MES buffer (pH 6.5, 80 mM) at 35°C (Jing *et al*., 2019). Assays were performed with *Ds*P4H and substrates of different concentrations. *V*_max_ and *K*_m_ were estimated from Michaelis–Menten model (Sheiner and Beal, 1980).

### Effects of temperature and pH on *Ds*P4H Activity

To determine the effect of temperature on *Ds*P4H activity, we measured its activity at a temperature range of 15–45°C with standard reaction mixture. The effect of pH on *Ds*P4H activity was determined by incubating standard reaction mixture to different pH ranging from 4.0 to 9.0.

### Enzymes activity assays

P4H activity was assayed as previously reported by (Yi *et al*., 2014). The reaction mixture contained 80 mM MES buffer (pH 6.5), 4 mM l‐proline, 8 mM α‐KG, 2 mM FeSO_4_, 4 mM l‐ascorbic acid and cells (or the purified P4H). This mixture was incubated at 35°C for 10 min with shaking, and then, cellular activity was inactivated completely by heat treatment at 100°C for 5 min. Hyp concentration in this mixture was measured after centrifugation. One unit of P4H activity was defined as the amount of enzyme that forms 1 nmol of Hyp in one minute.

### Production of Hyp from l‐proline in shake flasks

The transformation reaction was optimized under the following conditions for 16 h in 500 ml flasks at 35°C with rotation at 200 r.p.m: 50 ml MES buffer (80 mM, pH 6.5), 30 g l^−1^ l‐proline, 50 g l^−1^ α‐KG, 15 g l^−1^ wet cell weight, 0.3 g l^−1^ FeSO_4_, 1.0 (v/v) % Triton X‐100, 0.7 g l^−1^ l‐ascorbic acid.

### Production of Hyp from L‐proline in bioreactors

The optimal transformation reaction was scaled up for Hyp production in 5‐l bioreactors with a 3‐l working volume for 36 h at 35°C, 200 r.p.m.: 3 l MES buffer (80 mM, pH 6.5), 50 g l^−1^ l‐proline, 20 g l^−1^ α‐KG, 20 g l^−1^ wet cell weight, 0.5 g l^−1^ FeSO_4_, 1.5 (v/v) % Triton X‐100, 0.7 g l^−1^ l‐ascorbic acid. 50 g l^−1^ l‐proline and 20 g l^−1^ α‐KG were fed at 16 h.

### Statistical analysis

All measurements were taken in triplicate, and experiments were repeated three times to calculate the standard deviation.

## Conflict of interest

The authors declare no conflict of interest.
